# Combined conventional factors and the radiomics signature of coronary plaque texture could improve cardiac risk prediction

**DOI:** 10.1186/s13244-024-01759-9

**Published:** 2024-07-06

**Authors:** Jannik Kahmann, Dominik Nörenberg, Theano Papavassiliu, Salman Ul Hassan Dar, Sandy Engelhardt, Stefan O. Schoenberg, Matthias F. Froelich, Isabelle Ayx

**Affiliations:** 1 Department of Radiology and Nuclear Medicine, University Medical Centre Mannheim, Heidelberg University, Theodor-Kutzer-Ufer 1-3, 68167 Mannheim, Germany; 2 First Department of Internal Medicine, University Medical Centre Mannheim, Medical Faculty Mannheim, University of Heidelberg, 68167 Mannheim, Germany; 3 Department of Internal Medicine III, Heidelberg University Hospital, Heidelberg, Germany; 4 AI Health Innovation Cluster, Heidelberg, Germany

**Keywords:** Tomography (X-ray computed), Coronary artery disease, Epicardial adipose tissue, Radiomics

## Abstract

**Objectives:**

This study aims to investigate how radiomics analysis can help understand the association between plaque texture, epicardial adipose tissue (EAT), and cardiovascular risk. Working with a Photon-counting CT, which exhibits enhanced feature stability, offers the potential to advance radiomics analysis and enable its integration into clinical routines.

**Methods:**

Coronary plaques were manually segmented in this retrospective, single-centre study and radiomic features were extracted using pyradiomics. The study population was divided into groups according to the presence of high-risk plaques (HRP), plaques with at least 50% stenosis, plaques with at least 70% stenosis, or triple-vessel disease. A combined group with patients exhibiting at least one of these risk factors was formed. Random forest feature selection identified differentiating features for the groups. EAT thickness and density were measured and compared with feature selection results.

**Results:**

A total number of 306 plaques from 61 patients (mean age 61 years +/− 8.85 [standard deviation], 13 female) were analysed. Plaques of patients with HRP features or relevant stenosis demonstrated a higher presence of texture heterogeneity through various radiomics features compared to patients with only an intermediate stenosis degree. While EAT thickness did not significantly differ, affected patients showed significantly higher mean densities in the 50%, HRP, and combined groups, and insignificantly higher densities in the 70% and triple-vessel groups.

**Conclusion:**

The combination of a higher EAT density and a more heterogeneous plaque texture might offer an additional tool in identifying patients with an elevated risk of cardiovascular events.

**Clinical relevance statement:**

Cardiovascular disease is the leading cause of mortality globally. Plaque composition and changes in the EAT are connected to cardiac risk. A better understanding of the interrelation of these risk indicators can lead to improved cardiac risk prediction.

**Key Points:**

- Cardiac plaque composition and changes in the EAT are connected to cardiac risk.
- Higher EAT density and more heterogeneous plaque texture are related to traditional risk indicators.
- Radiomics texture analysis conducted on PCCT scans can help identify patients with elevated cardiac risk.

**Graphical Abstract:**

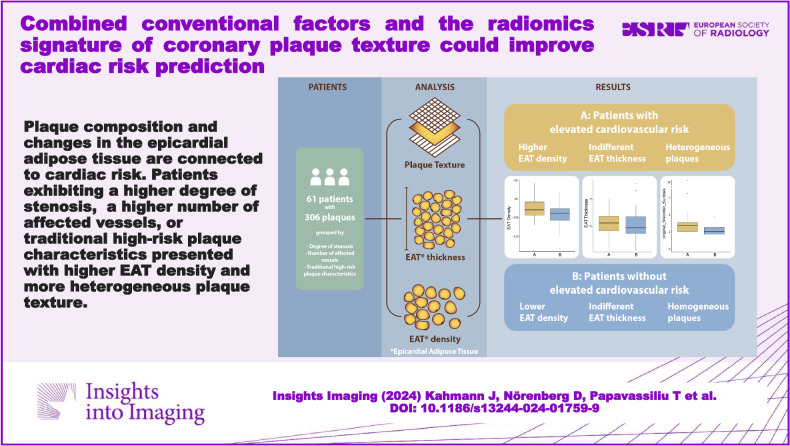

## Introduction

The treatment and prevention of cardiovascular disease (CVD), the leading cause of morbidity and mortality globally [1], remains one of modern healthcare’s greatest challenges. The risk for severe cardiovascular events differs significantly depending on individual risk factors including the degree of stenosis caused by the plaques, special high-risk compositions of plaques, and a high number of plaques or affected coronary vessels [2, 3].

High-risk plaque (HRP) features include spotty calcification, positive remodelling, low attenuation, and the napkin-ring sign [4]. Plaques with these characteristics have an elevated risk of causing cardiovascular events through plaque rupture or erosion [5, 6]. Nevertheless, the vulnerability of HRP is a fluctuating state influenced by other factors like biomechanics and inflammatory states [6]. Therefore, the need emerges to look deeper into HRP texture and associate it with additional risk-indicating factors.

Recent studies outlined an interesting connection between epicardial adipose tissue (EAT) and the pathogenesis of CVD [7, 8]. In this context, EAT density and volume have been of particular interest [9]. EAT and the underlying myocardium share the same microcirculation and are in immediate bidirectional interaction with each other [10]. While EAT under physiological conditions can have a protective effect on the heart [10], it can also advance the development of CVD through inflammation, exaggerated immunity response, oxidative stress, and glucotoxicity [11]. In addition, EAT volume has been linked to the presence of HRP [12]. Since EAT is responsive to certain drugs with positive effects on the occurrence of major adverse cardiovascular events (MACE) [13, 14], understanding this important tissue can be extremely beneficial.

Coronary computed tomography angiography (CCTA) is the diagnostic choice for patients with a moderate pretest likelihood of CVD [15]. It offers comparable diagnostic efficacy while minimising procedure-related complications in contrast to initial invasive coronary angiography [16]. Traditionally, CCTA relies on the subjective assessment of qualitative visual features, often dependent on the examiner’s expertise. However, significant progress in cardiac computed tomography (CT) has enabled the evaluation of tissue texture through radiomics analysis [17], which involves quantifying pixel intensity patterns within an image, thereby providing valuable insights into tissue heterogeneity, composition, and structural characteristics [18]. Realising the full potential of radiomics texture analysis requires addressing specific challenges that currently hinder its final integration into clinical protocols. Concerns persist regarding its susceptibility to variations in technical parameters, such as reconstruction algorithms, contrast, and layer thickness [19, 20].

In contrast to conventional energy-integrating CT (EICT) devices, the novel photon-counting CT (PCCT) technology employs smaller photon-counting detector elements. These photon-counting detector elements directly convert every incoming photon hitting the detector plate into an electrical impulse [21], equipping PCCT with superior spatial resolution, enhanced signal-to-noise ratio, and reduced beam hardening artefacts [21, 22]. It thereby offers improved feature stability, providing the possibility for advancing radiomics analysis and addressing several of the limitations [23, 24].

This study aimed to investigate differentiating characteristics in coronary plaque texture in patients presenting with traditional risk factors for a heightened risk of cardiovascular events and to compare it to traditional factors and differences in EAT thickness and density to provide a potential imaging biomarker for cardiovascular risk assessment.

## Methods

### Study design

This study followed the Declaration of Helsinki principles and received approval from the institutional review board and local ethics committee (ID 2021-659). From April to July 2022, this retrospective single-centre study included patients meeting clinical criteria for contrast-enhanced cardiac CT per European Society of Cardiology guidelines [25] and who exhibited the detection of at least one coronary plaque in cardiac CT. None of these patients had a history of ischaemic cardiac disease. Exclusions applied to those with a prior pacemaker or cardiac stent implantation or severe image artefacts. Clinical parameters were retrospectively obtained from an existing traditional clinical risk factors questionnaire.

### Patient collective and plaque distribution

In total, 61 patients (13 female, 48 male, mean age 61 years, range: 40–82 years) were selected according to inclusion and exclusion criteria. The patients presented with a total of 306 plaques (265 calcified, 19 non-calcified, 22 mixed). First, all patients who presented with HRP were identified. HRP was defined as plaques expressing at least one of the following high-risk features: spotty calcification, positive remodelling, low attenuation, and napkin-ring sign [4]. Three additional factors associated with an elevated risk of cardiovascular events were determined: plaques with at least 50% stenosis, plaques with at least 70% stenosis, or triple-vessel coronary artery disease (regardless of the degree of stenosis) [2, 3]. The patient group was subsequently divided into multiple subgroups (“HRP”, “50%”, “70%”, and “triple-vessel” group) based on the presence or absence of these factors. Furthermore, the patient population was categorised into two main groups: one consisting of individuals who met at least one of the elevated risk factor criteria (“combined group”) and another consisting of patients who did not exhibit any of these risk factors (Table 1).

**Table 1:** Patient collective overview. Mean and (SD) are given for continuous variables

Patient characteristics	Overall	50% Stenosis	70% Stenosis	HRP	Triple-vessel	Combined
*n*	61	16	5	22	19	32
Age	60.95 (8.85)	61.50 (8.68)	59.40 (4.08)	60.00 (8.83)	62.11 (10.40)	60.84 (8.94)
Sex m/f	48/13	16/0	5/0	21/1	18/1	31/1
Agatston Score	261.70 (618.28)	709.84 (1059.83)	1350.84 (1704.49)	317.10 (109.31)	362.2 (225.33)	422.78 (809.25)
Plaques (calcified, non-calcified, mixed)	306 (265, 19, 22)	171 (151, 5, 15)	77 (64, 5, 8)	165 (136, 11, 18)	175 (155, 5, 15)	253 (219, 14, 20)

### Cardiac CT imaging

All 61 patients were examined using a first-generation whole-body dual-source PCCT system (NAEOTOM Alpha; Siemens Healthcare GmbH, Forchheim, Germany). The examination utilised a prospective electrocardiographic (ECG)-gated sequential mode with a tube voltage of 120 kV and automatic dose modulation. The CARE keV BQ setting was configured at 64, and the gantry rotation time was 0.25 s.

To maintain heart rates below 65 beats per minute, patients received intravenous β-blockers in the 5–10 mg range, provided there were no contraindications, and the dosage was adjusted based on individual heart rates. Following, sublingual nitroglycerin (0.8 mL) was administered. A non-enhanced cardiac CT scan was conducted to evaluate coronary artery calcification. Subsequently, a contrast-enhanced scan was performed using 80 mL of iodine contrast (Imeron 400, Bracco Imaging Deutschland GmbH, Konstanz, Germany), accompanied by a 20 mL saline chaser (NaCl 0.9%) at a weight-based flow rate of 5–6 mL/sec. CCTA was initiated by bolus tracking in the ascending thoracic aorta.

### Cardiac CT imaging analysis

Coronary artery calcification assessment involved non-enhanced axial scans with a slice thickness of 3 mm and Qr36 kernel, which were processed using dedicated syngo.via software (Siemens Healthcare GmbH, Forchheim, Germany) to estimate the degree of coronary artery calcification using the Agatston score. In addition, axial contrast-enhanced CCTA images were reconstructed with a slice thickness of 0.6 mm and an increment of 0.4 mm, employing a soft vascular kernel (Bv40).

Subsequently, the contrast-enhanced images underwent an anonymisation process and were exported as Digital Imaging and Communications in Medicine files. They were then transformed into Neuroimaging Informatics Technology Initiative format and then imported into 3D Slicer (Version 4.11), a specialised segmentation tool [26].

The evaluation of coronary arteries in terms of plaque morphology and the extent of stenosis was carried out by a senior radiologist possessing over a decade of experience in cardiothoracic imaging. All 306 coronary plaques were segmented manually by a medical student with over one year of experience in image segmentation and validated by the same senior radiologist.

**Fig. 1:**
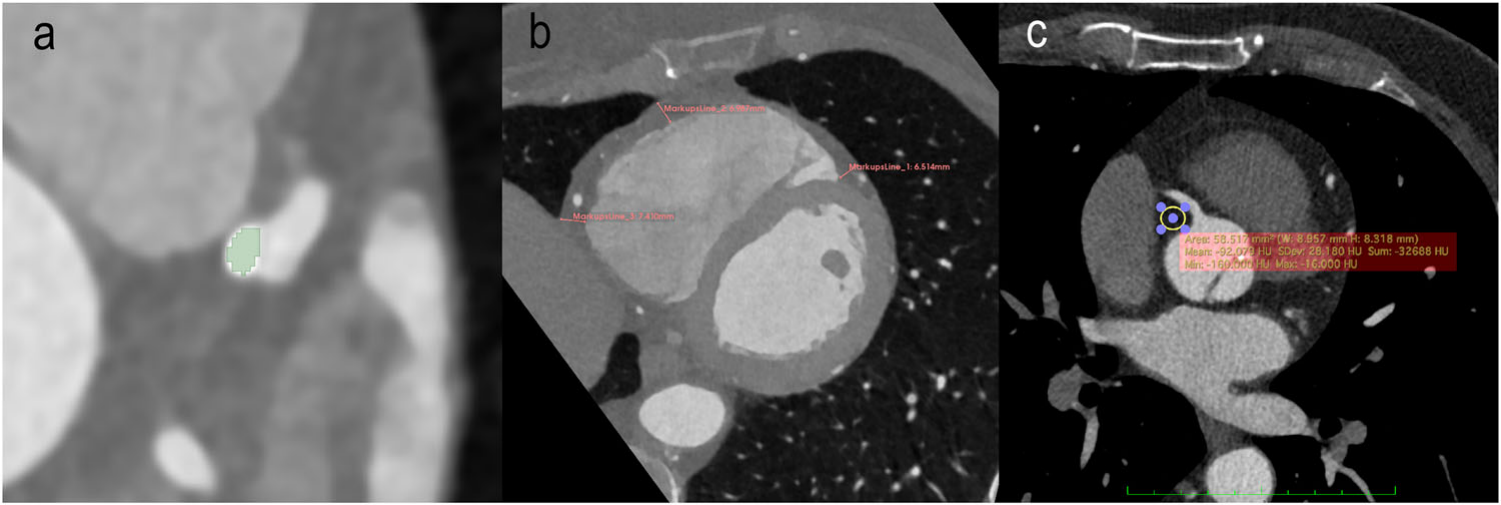
**a** Example segmentation of a coronary plaque (in green) in a 63-year-old male. **b** Example measurement of EAT thickness in a 69-year-old female. **c** Example measurement of EAT density in a 64-year-old male

In addition, the EAT density (reported in Hounsfield units (HU)) and mean thickness (reported in mm) of every patient were measured. EAT density was measured using virtual monoenergetic reconstructions at 70 keV as recommended in literature with a slice thickness of 0.6 mm, an increment of 0.4 mm, and a soft vascular kernel (Qr40) [27]. Using Horos PACS (Version 3.3.6), a region of interest (ROI) was set in the EAT next to the origin of the right coronary artery on a single slice and the mean, minimum and maximum density were measured. The ROI size was chosen as large as possible while avoiding adjacent structures [28]. EAT thickness was assessed after reconstruction along the short axis of the heart by calculating the mean of three distinct measurements from the outer myocardium to the visceral epicardium at the basal level of the right ventricular anterior free wall [29]. Figure 1 shows an example segmentation of a coronary plaque and example measurements for EAT density and thickness.

### Radiomics feature extraction and statistical analysis

Using pyradiomics (version 3.0.1) [30], radiomics features, including shape, first-order, Grey Level Co-occurrence Matrix, Grey Level Dependence Matrix, Grey Level Size Zone Matrix, Grey Level Run Length Matrix, and Neighbouring Grey Tone Difference Matrix, were extracted. The texture features were imported into R Statistics (Version 4.2.0, R Core Team, Vienna, Austria) [31] for further analysis, conducted in RStudio (Version 2022.07.1 + 554, Boston, MA) [32]. Mean and standard deviation (SD) values were computed. All radiomics features underwent normalisation using the *z*-score formula:

$$z=((X-\mu ))/\sigma$$

A clustered heatmap, visually representing the distribution of these extracted features, was generated using the ComplexHeatmap package for R. A permutation-based random forest (RF) classification with the Boruta package for R was conducted for every subset of patients. Boxplots of the selected features were generated for visualisation.

### Combination of radiomics features and EAT density

Two scatter plots were created, mapping the radiomics features “original_firstorder_Kurtosis” and “original_glrlm_RunLengthNonUniformityNormalized” to the EAT mean density. Within these plots, patients of the combined group were compared to their counterparts.

## Results

### Plaque assessment

A total of 306 plaques were segmented in the 61 patients enroled in this study. Two hundred and sixty-five of these plaques were completely calcified, 19 were non-calcified, and 22 were partially calcified/mixed. On average, patients presented with five plaques (range: 1–26). The distribution of the plaques within the patient subsets was as follows: 171 plaques in 16 patients (average: 10.69) in the 50% group, 77 plaques in five patients (average: 15.40) in the 70% group, 165 plaques in 22 patients (average: 7.50) in the HRP group, 175 plaques in 19 patients (average: 9.21) in the triple-vessel group, and 253 plaques in 32 patients (average: 7.91) in the combined group (Table 1).

### EAT comparison

**Fig. 2:**
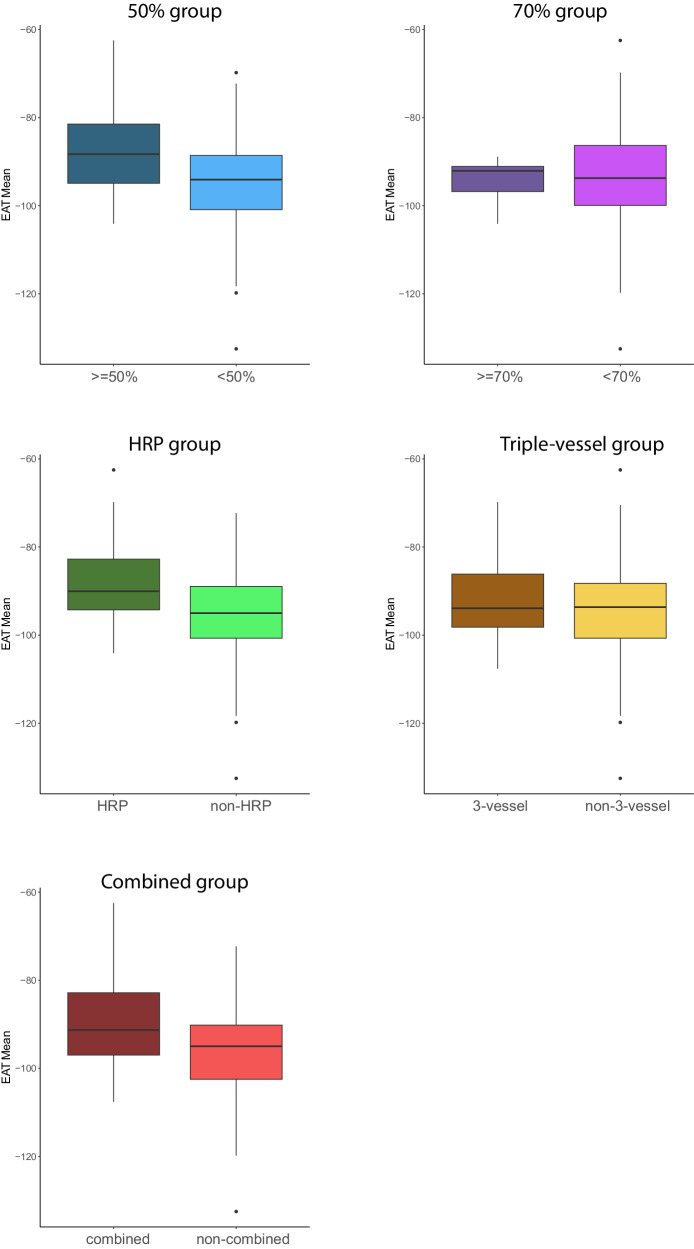
EAT mean density of the 50%, 70%, HRP, 3-vessel, and combined group

EAT thickness and density of all five groups were investigated and compared to the patients not exhibiting any of the risk factors (non-combined group). A *p*-value below 0.05 was considered significant. None of the groups differed significantly from the non-combined group regarding EAT thickness (*p* = 0.222 – *p* = 0.877). Regarding EAT density, mainly significant differences between the groups were found. All groups showed a higher mean EAT density than non-affected patients: −97.55 HU for the non-combined group to −87.28 HU (*p* = 0.008) for the 50% group, −94.60 HU (*p* = 0.424) for the 70% group, −87.92 HU (*p* = 0.006) for the HRP, −91.27 HU (*p* = 0.066) for the triple-vessel, and −89.35 HU (*p* = 0.008) for the combined group. Except for the triple-vessel and the 70% group, these differences were all significant. The minimum and maximum density followed the same tendencies. Only the minimum density of the 70% group was slightly lower compared to the non-combined group (Table 2). The EAT’s mean densities for all groups are visualised as boxplots (Fig. 2).

**Table 2:** EAT data overview

	Overall	Non-combined	50%	70%	HRP	Triple-vessel	Combined
EAT thickness	10.44 (2.55)	10.51 (2.90)	10.80 (2.54) [*p* = 0.730]	11.57 (1.38) [*p* = 0.222]	10.14 (2.27) [*p* = 0.610]	10.62 (2.00) [*p* = 0.877]	10.37 (2.24) [*p* = 0.833]
Mean density	−93.25 (12.23)	−97.55 (12.50)	−87.28 (11.37) [*p* = 0.008]	−94.60 (6.04) [*p* = 0.424]	−87.92 (11.51) [*p* = 0.006]	−91.27 (10.43) [*p* = 0.066]	−89.35 (10.73) [*p* = 0.008]
Min density	−165.44 (23.08)	−173.69 (24.90)	−158.69 (19.96) [*p* = 0.034]	−174.40 (17.18) [*p* = 0.939]	−154.95 (18.20) [*p* = 0.003]	−158.68 (19.30) [*p* = 0.024]	−157.97 (18.69) [*p* = 0.007]
Max density	−22.31 (16.24)	−27.55 (17.54)	−13.25 (10.13) [*p* = 0.001]	−11.20 (4.44) [*p* = 0.0002]	−17.18 (13.32) [*p* = 0.020]	−20.05 (12.98) [*p* = 0.096]	−17.56 (13.55) [*p* = 0.015]

Every group is compared to the group of patients that do not exhibit any of the risk factors (non-combined). Mean and (SD) are given for continuous variables

### Cluster analysis

**Fig. 3:**
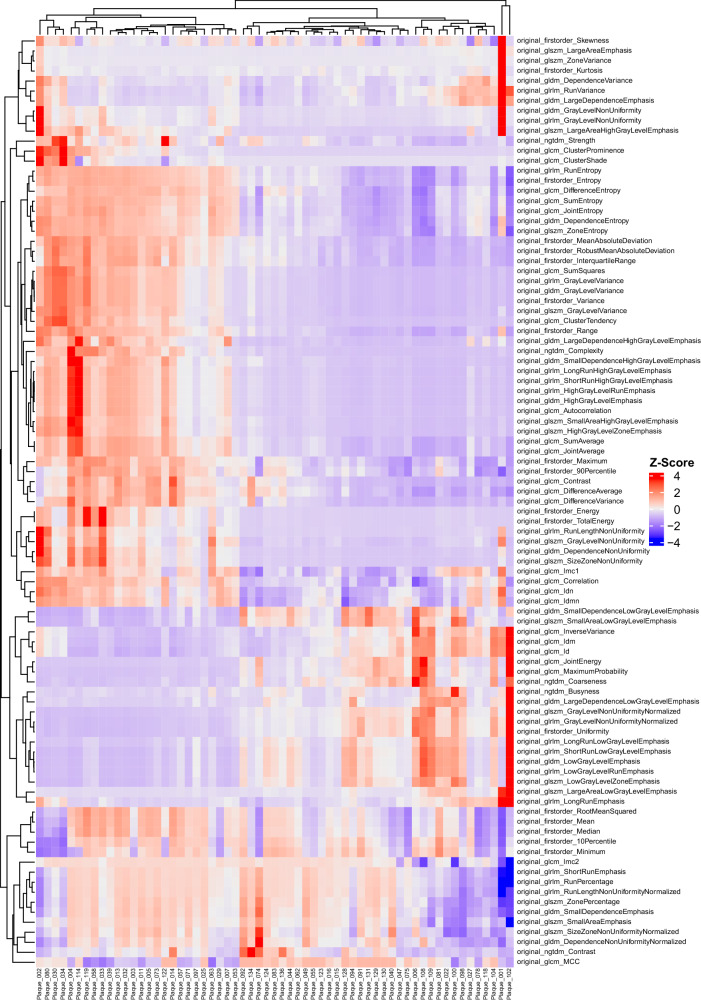
Unsupervised clustering heatmap of coronary plaque radiomics features

The extracted radiomics features of all patients were subjected to unsupervised k-means clustering. The resulting heatmap illustrates a separation of patients into two main clusters (Fig. 3). A deeper investigation revealed a notable difference exclusively in the Agatston scores. The 26 patients forming the left cluster presented with a mean Agatston score of 455.25, while the mean of the 35 patients in the right cluster was 113.69.

### Feature selection

**Fig. 4:**
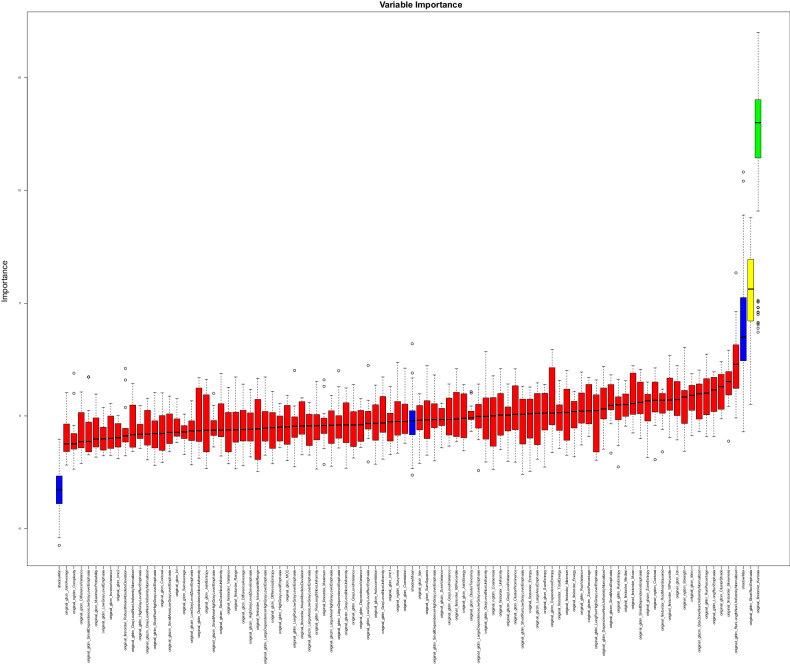
Random forest (RF) feature selection for the combined group: features relevant to the differentiation are in green

**Fig. 5:**
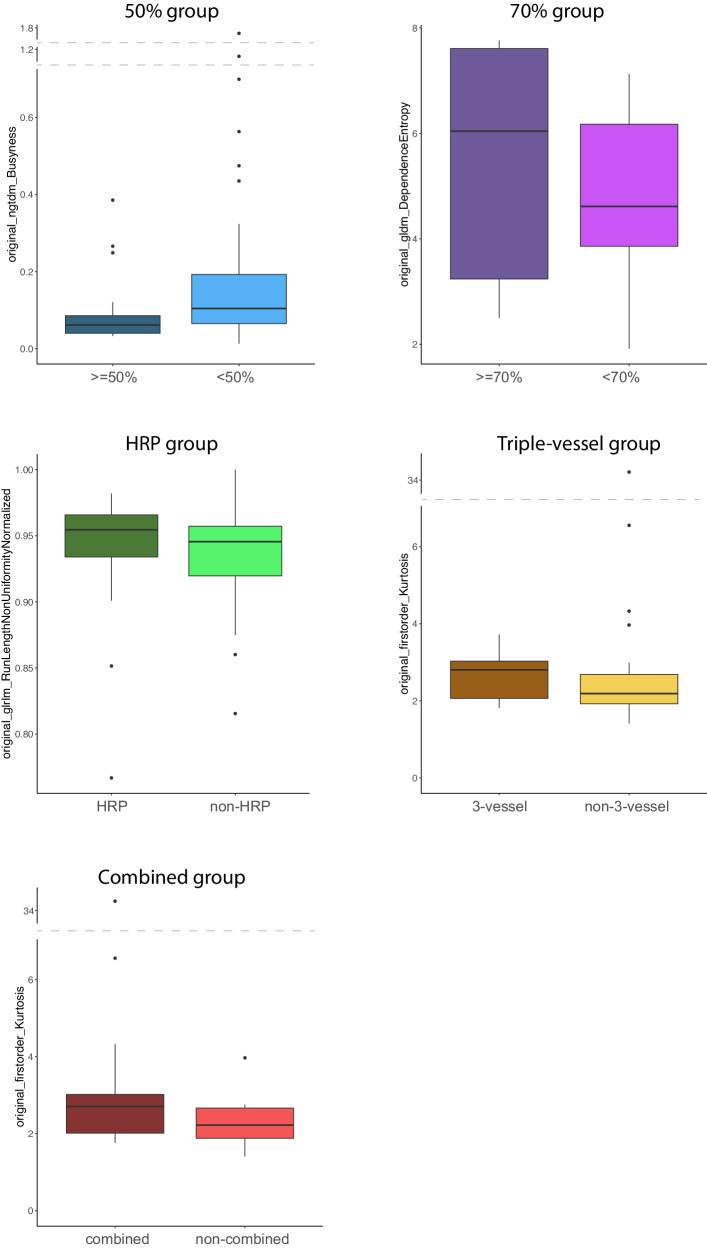
Distribution of the most important selected features within the respective patient subsets

Texture features aiding in distinguishing between patient groups were identified using RF feature selection. This selection process was carried out for all the subgroups and the combined group. The features “original_ngtdm_Busyness” and “original_glcm_MaximumProbability” were found to distinguish between patients with and without at least 50% stenosis, while patients of the 70% group could be discriminated by the features “original_glcm_Idmn”, “original_gldm_DependenceEntropy”, and “original_glszm_ZoneEntropy”. For identifying the triple-vessel group, the features “original_glrlm_RunPercentage”, “original_glrlm_LongRunEmphasis”, and “original_firstorder_Kurtosis” were selected. Regarding the HRP group, only the feature “original_glrlm_RunLengthNonUniformityNormalized” was identified as distinctive. Differentiation of the combined group was made possible through the feature “original_firstorder_kurtosis”. (Fig. 4). A summary of all the selected features and their values in the respective groups is offered in Table 3 and the most important selected features from each group are presented as boxplots (Fig. 5).

**Table 3:** Selected features of every patient subset

Group	Feature	Value – group	Value – counterpart	Value – non-combined
50%	ngtdm_Busyness	0.10 (0.10)	0.21 (0.32)	0.23 (0.38)
	glcm_MaximumProbability	0.08 (0.12)	0.10 (0.12)	0.11 (0.14)
70%	glcm_Idmn	0.94 (0.04)	0.91 (0.04)	0.91 (0.04)
	gldm_DependenceEntropy	5.43 (2.45)	4.82 (1.39)	4.60 (1.39)
	glszm_ZoneEntropy	5.13 (2.28)	4.61 (1.35)	4.39 (1.37)
HRP	glrlm_RunLengthNonUniformityNormalized	0.94 (0.05)	0.93 (0.04)	0.94 (0.04)
Triple-vessel	glrlm_RunPercentage	0.97 (0.02)	0.96 (0.03)	0.97 (0.02)
	glrlm_LongRunEmphasis	1.09 (0.06)	1.14 (0.14)	1.13 (0.13)
	firstorder_Kurtosis	2.64 (0.56)	3.19 (4.99)	2.30 (0.51)
Combined	firstorder_Kurtosis	3.67 (5.67)	2.30 (0.51)	2.30 (0.51)

Mean and (SD) are given for continuous variables

*GLCM* grey level co-occurrence matrix, *GLDM* grey level dependence matrix, *GLRLM* grey level run length matrix, *GLSZM* grey level size zone matrix, *NGTDM* neighbouring grey tone difference matrix

### Association of heterogeneity, EAT density, and elevated cardiovascular risk

**Fig. 6:**
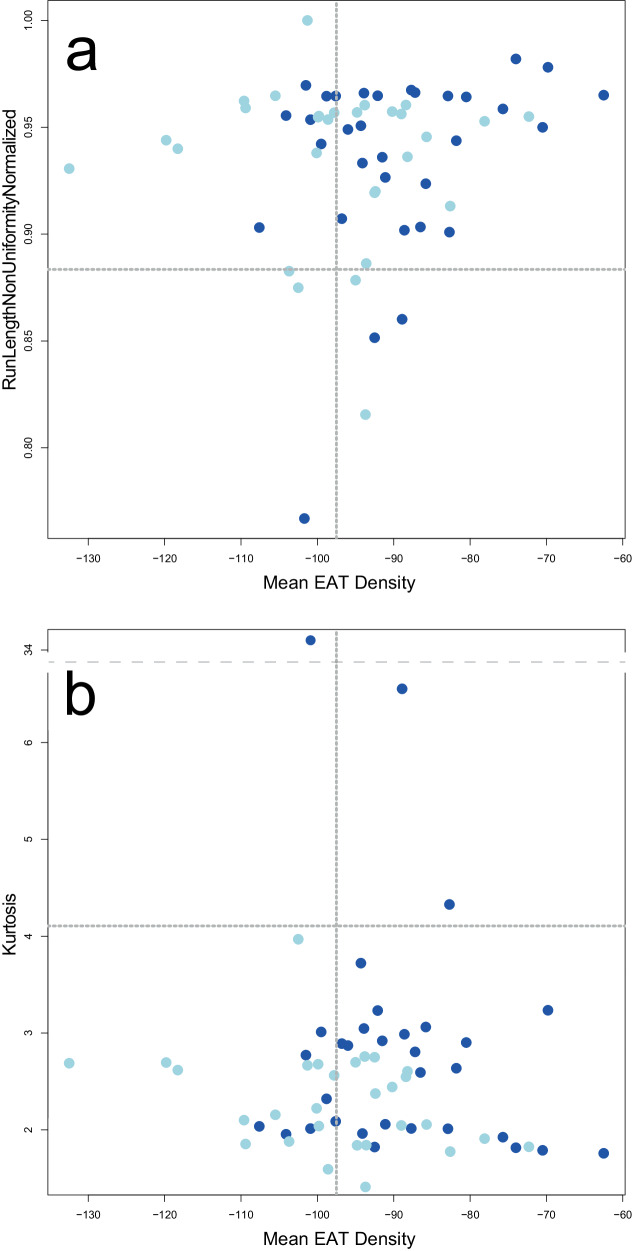
Comparison of heterogeneous plaque texture features and EAT density in patients of the combined group (dark blue) and its counterpart. **a** “original_glrlm_RunLengthNonUniformityNormalized”, **b** “original_firstorder_Kurtosis”

Two scatter plots were generated, illustrating the relationship between radiomics features (“original_firstorder_Kurtosis” and “original_glrlm_RunLengthNonUniformityNormalized”) and the mean density of EAT. The comparison focused on patients in the combined group versus their counterparts. These plots illustrate how patients in the combined group with an elevated risk for cardiovascular events overall presented with a higher EAT mean density and more heterogeneous plaque texture according to the selected features (Fig. 6).

## Discussion

This study demonstrates how radiomics feature analysis of coronary plaque texture can offer a way to detect plaques correlated with an elevated risk for cardiovascular events. The analysis of EAT thickness did not reveal any meaningful differences. Investigation of EAT density produced significant differences for the 50% group, the HRP group, and the combined group with a higher density in affected patients. However, for the two groups, no significant difference could be described, which emphasises the uncertainty about the relation between EAT density and an elevated cardiovascular risk of coronary artery disease that persists in current literature [33]. This dissent may be countered by developing and integrating a radiomics signature of coronary plaque texture into the risk prediction process.

The radiomics profile outlined a more heterogenous plaque texture in patients with an elevated risk of cardiovascular events. The HRP group and the combined group could be best discriminated by a more heterogenous plaque texture. In line with these findings, two out of three relevant discriminating features indicate a more heterogeneous texture of the plaque in the 70% group. On the other hand, patients with only intermediate coronary artery stenosis showed more homogenous plaque structures and could not be differentiated by heterogeneity. Hence, this study outlines the correlation of a more heterogeneous plaque texture with conventional risk factors of cardiac plaques. In addition, the combination of EAT density and heterogenous plaque texture might offer an imaging biomarker for cardiovascular risk prediction in the future.

Numerous studies investigated the correlation between EAT thickness, EAT density, and CVD or elevated risk for cardiovascular events. However, some inconsistencies in their findings exist.

Goeller et al investigated if CVD, plaque inflammation, and MACE are connected to EAT density and volume. Their study involving non-contrast cardiac CT scans of 456 asymptomatic patients found EAT volume to be higher and EAT density to be lower in individuals presenting with coronary calcium compared to those without. EAT volume was also positively correlated with the degree of atherosclerosis. In addition, lower EAT density and higher volume were significantly associated with MACE [34]. While some studies are in line with these results [35, 36], others come to different conclusions.

A study by Pracon et al reported an association between increased EAT density and coronary atherosclerosis defined as CVD or a positive Agatston score. A total of 164 patients underwent coronary angiography, 36 of whom presented with CVD. These patients showed significantly higher EAT density than subjects without CVD (−78.99 ± 4.12 vs. −81.57 ± 4.64 HU, *p* < 0.01). Furthermore, the density was positively correlated with the patients’ Agatston score (*r* = 0.23, *p* < 0.01) [37].

Regarding EAT density, our results mostly agree with the studies reporting an increased density in subjects with a higher degree of CVD or elevated cardiovascular risk. However, the assessment of EAT thickness did not produce any significant differences. This reflects the inconsistency found in the literature and points towards the possible necessity of expanding the focus and including coronary plaque texture features in the risk assessment process.

Furthermore, the coronary plaque texture has been subject to recent studies. To predict MACE over a median three-year follow-up, Chen et al developed a radiomic plaque signature involving 14 textural features and two shape features. The retrospective study included a radiomic signature development set consisting of 225 patients with 419 plaques. In conclusion, a high radiomic signature was independently associated with the incidence of MACE (hazard ratio = 2.01; *p* = 0.005) [38]. These promising results should be motivation to further investigate coronary plaque texture to understand its association with other available risk predictors and develop a way to combine the radiomic signature with traditional risk factors.

A retrospective study by Tobe et al involving 691 patients who underwent percutaneous coronary intervention and carotid ultrasound, aimed to explore the relationship between carotid artery ultrasound findings and clinical outcomes. The maximum carotid intima-media thickness (CIMT) and characteristics of carotid plaques were assessed visually. Patients with heterogeneous carotid plaques (maximum CIMT ≥ 1.5 mm and heterogeneous texture) were found to be at a higher risk of MACE, suggesting the need for more aggressive medical therapy and vigilant follow-up in these patients [39]. While this investigation’s results are based on different diagnostic modalities, the findings are in line with our study, strengthening the suggestion that a heterogeneous plaque texture indicates a heightened cardiovascular risk.

Nonetheless, certain limitations persist in this study. The approach as a single-centre study and the relatively modest size of the study cohort were due to the very novel implementation of the PCCT scanner. Consequently, the reproducibility challenge associated with radiomics analysis was not thoroughly addressed. Vital steps in translating our findings into clinical practice involve standardising texture analysis methodologies and ensuring reproducibility across various PCCT platforms. Particularly the 70% group only consisted of five patients, thereby possibly making it susceptible to overfitting by the RF feature selection algorithm. Nevertheless, other inquiries outlined that PCCT offers deeper insights into texture variations in comparison to EICT [40]. The enhanced stability of radiomics features observed in the PCCT context, suggests a promising avenue for improving comparability through PCCT implementation [23]. Future studies should focus on using a prospective multicentre approach involving a more extensive study population with sufficient clinical data to address these limitations.

In conclusion, structural differences in coronary plaque texture, mainly a higher heterogeneity, were found in patients with an elevated risk for cardiovascular events. EAT density seemed to be higher in patients expressing these risk factors, while no significant change in EAT thickness could be detected. Using advanced imaging techniques and modern computational analysis to integrate a combination of the radiomics plaque texture signature and EAT attenuation into traditional risk factors may promise to further improve cardiac risk assessment and management in the future.

## Data Availability

The datasets generated during and/or analysed during the current study are available from the corresponding author on reasonable request.

## Abbreviations

CCTA: Coronary computed tomography angiography
CIMT: Carotid intima-media thickness
CT: Computed tomography
CVD: Cardiovascular disease
EAT: Epicardial adipose tissue
EICT: Energy-integrating detector CT
HRP: High-risk plaques
HU: Hounsfield units
MACE: Major adverse cardiovascular events
PCCT: Photon-counting computed tomography
RF: Random forest
SD: Standard deviation
